# NT-proBNP as a Potential Marker of Cardiovascular Damage in Children with Chronic Kidney Disease

**DOI:** 10.3390/jcm10194344

**Published:** 2021-09-24

**Authors:** Piotr Skrzypczyk, Magdalena Okarska-Napierała, Radosław Pietrzak, Katarzyna Pawlik, Katarzyna Waścińska, Bożena Werner, Małgorzata Pańczyk-Tomaszewska

**Affiliations:** 1 Department of Pediatrics and Nephrology, Medical University of Warsaw, 02-091 Warsaw, Poland; magda.okarska@gmail.com (M.O.-N.); mpanczyk1@wum.edu.pl (M.P.-T.); 2 Department of Pediatrics with Clinical Assessment Unit, Medical University of Warsaw, 02-091 Warsaw, Poland; 3 Department of Paediatric Cardiology and General Paediatrics, Medical University of Warsaw, 02-091 Warsaw, Poland; radoslaw.pietrzak@wum.edu.pl (R.P.); bozena.werner@wum.edu.pl (B.W.); 4 Student Scientific Group at the Department of Pediatrics and Nephrology, Medical University of Warsaw, 02-091 Warsaw, Poland; katarzynapawlik97@gmail.com (K.P.); s068819@student.wum.edu.pl (K.W.)

**Keywords:** chronic kidney disease, NT-proBNP, children, cardiovascular disease, common carotid artery intima-media thickness

## Abstract

Assessing cardiovascular disease (CVD) in children with chronic kidney disease (CKD) is difficult. Great expectations have been associated with biomarkers, including the N-terminal pro-brain natriuretic peptide (NT-proBNP). This study aimed to determine the correlation between NT-proBNP and cardiovascular complications in children with CKD. Serum NT-proBNP, arterial stiffness, common carotid artery intima-media thickness (cIMT), echocardiographic (ECHO) parameters (including tissue Doppler imaging), and biochemical and clinical data were analyzed in 38 pediatric patients with CKD (21 boys, 12.2 ± 4.2 years). Mean NT-proBNP in CKD patients was 1068.1 ± 4630 pg/mL. NT-proBNP above the norm (125 pg/mL) was found in 16 (42.1%) subjects. NT-proBNP correlated with glomerular filtration rate (GFR) (r = −0.423, *p* = 0.008), and was significantly higher in CKD G5 (glomerular filtration rate grade) patients compared to CKD G2, G3, and G4 children (*p* = 0.010, *p* = 0.004, and *p* = 0.018, respectively). Moreover, NT-proBNP correlated positively with augmentation index (AP/PP: r = 0.451, *p* = 0.018, P2/P: r = 0.460, *p* = 0.016), cIMT (r = 0.504, *p* = 0.020), and E/E’ in ECHO (r = 0.400, *p* = 0.032). In multivariate analysis, logNT-proBNP was the only significant predictor of cIMT Z-score (beta = 0.402, 95CI (0.082–0.721), *p* = 0.014) and P2/P1 (beta = 0.130, 95CI (0.082–0.721), *p* = 0.014). Conclusions: NT-proBNP may serve as a possible marker of thickening of the carotid artery wall in pediatric patients with CKD. The final role of NT-proBNP as a biomarker of arterial damage, left ventricular hypertrophy, or cardiac diastolic dysfunction in CKD children needs confirmation in prospective studies.

## 1. Introduction

Children with chronic kidney disease (CKD) have been recognized as the pediatric group with the highest risk of cardiovascular disease (CVD) [1]. Assessment of cardiovascular risk in children with CKD is difficult, as early stages of CVD do not cause symptoms and can progress undetected [2]. Direct evaluation of subclinical target organ damage in children with CKD requires expensive and not widely accessible devices, experienced and skilled personnel, is time-consuming and, commonly, operator-dependent. Hence, research has been conducted, aimed at finding serological markers of increased cardiovascular burden. Great expectation has been associated with the N-terminal pro-brain natriuretic peptide or the pro-B-type natriuretic peptide (NT-proBNP). As a response to increased left ventricular wall stretch due to volume overloads, and to structural damage of the myocardium, there is an increased expression of a proBNP in myocardial cells [3]. After cleavage to BNP and non-active NT-proBNP, both these particles are released to the bloodstream. Then NT-proBNP is excreted in urine without being metabolized further, while BNP can be captured by natriuretic peptide receptor types A–C, where it exerts its actions or is inactivated by neutral endopeptidase [4,5]. Physiological actions of BNP include the impact on kidneys (dilation of afferent arteriole and constriction of efferent arteriole, relaxation of mesangial cells, increased blood flow through vasa recta, decreased sodium reabsorption in the distal convoluted tubule and cortical collecting duct, inhibition of renin secretion), adrenal glands (reduction of aldosterone secretion), blood vessels (relaxation of vascular smooth muscles), myocardium (inhibition of maladaptive cardiac hypertrophy), and adipose tissue (release of free fatty acids) [4,6].

NT-proBNP is widely used to diagnose, screen, and stratify patients with heart failure and detect systolic and diastolic left ventricular dysfunction [3,7]. Its usefulness has already been investigated in adult CKD patients [8,9,10]. There are only scarce data on the prognostic value of BNP and NT-proBNP in pediatric patients with kidney impairment [11,12]. There are no data on the relationship between central blood pressure, arterial damage, and detailed echocardiographic evaluation and NT-proBNP in these children. Thus, this study aimed to determine the relationship between NT-proBNP and cardiovascular complications in children with CKD.

## 2. Materials and Methods

### 2.1. Study Group 

This single-center cross-sectional study involved 38 pediatric CKD subjects hospitalized during two years in one tertiary center of pediatric nephrology. The inclusion criteria were: age ≥ five years and CKD stages G2-5 (glomerular filtration rate grade) according to the Kidney Disease: Improving Global Outcomes (KDIGO) guidelines [13]. The following exclusion criteria were applied: coexisting cardiovascular diseases (e.g., congenital heart defects), treatment with recombinant human growth hormone, and acute infections (temporary exclusion for two weeks). 

**Figure 1:**
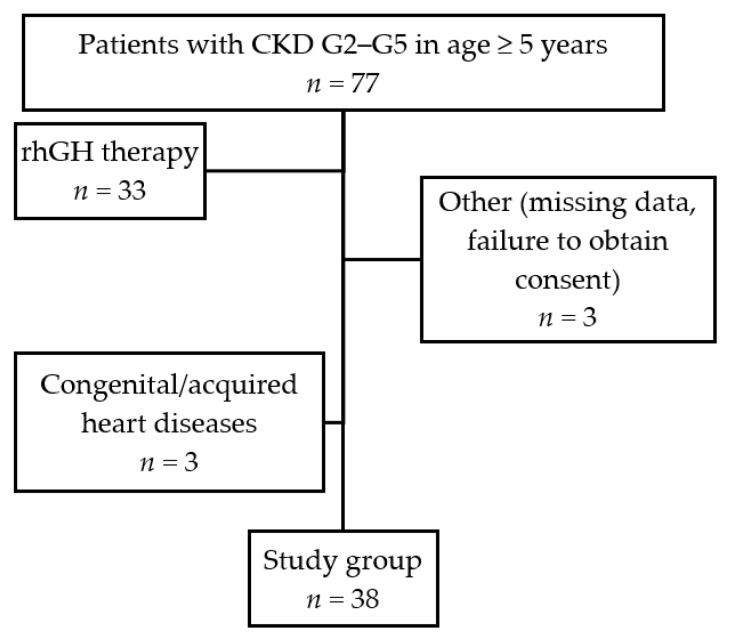
Flowchart of the patients’ recruitment (CKD—chronic kidney disease, G—Grade, rhGH—recombinant human growth hormone).

Participants were included in the study consecutively from among the patients after considering inclusion and exclusion criteria to exclude selection bias. The flowchart of the patients included in the study group is presented in Figure 1.

### 2.2. Ethical Issues

All procedures were in accordance with the ethical standards of the institutional review board (approval no. KB/89/2013) and with the 1964 Helsinki declaration and its later amendments. Informed consent was obtained from all legal representatives and individuals (≥16 years).

### 2.3. Clinical Parameters 

The following clinical data were collected: age (years), gender, CKD etiology [14], and stage [13] based on estimated glomerular filtration rate (GFR) [15], method of renal replacement therapy, body mass (kg), height [m] and body mass index (BMI) (kg/m^2^), Z-score [16], presence of arterial hypertension, and medications used.

### 2.4. NT-proBNP and Biochemical Parameters

Concentration of NT-proBNP (pg/mL) was determined in serum using the VITROS 5600 Integrated System (Ortho Clinical Diagnostics, Raritan, NJ, USA) with the upper limit taken from the manufacturer’s normative values (125 (pg/mL)). The following biochemical parameters were evaluated: creatinine (mg/dL), urea (mg/dL), uric acid (mg/dL), hemoglobin (g/dL), albumins (g/dL), calcium (mg/dL), inorganic phosphate (mg/dL), alkaline phosphatase (IU/mL), intact parathormone (pg/mL), 25-hydroxy-vitamin D (25(OH)D) (ng/mL), total, low-density (LDL) and high-density lipoprotein (HDL) cholesterol (mg/dL), triglycerides (mg/dL), and parameters of acid base balance from arterialized capillary blood: pH, and HCO_3_^−^ (mmol/L). All biochemical parameters were measured in the morning, on fasting, simultaneously. Normal values of hemoglobin and calcium–phosphorus metabolism parameters were taken from the Kidney Disease: Improving Global Outcome (KDIGO) guidelines [17,18], and the normal value of cholesterol and triglycerides from Stewart et al. [19]; hyperuricemia was recognized when uric acid was ≥5.5 (mg/dL) [20].

### 2.5. Blood Pressure and Parameters of Cardiovascular Damage

Peripheral office arterial blood pressure was measured with Welch Allyn VSM 300 Patient Monitor (Welch Allyn Inc., Skaneateles Falls, NY, USA) and expressed in (mmHg) and Z-score values [21]. Common carotid artery intima-media thickness (cIMT) was evaluated with a 13-MHz linear transducer (Aloka Prosound Alpha 6, Hitachi Aloka Medical, Mitaka, Japan), using methods described previously [22] and expressed in (mm) and Z-score [23]. Central blood pressure, arterial pulse waveform, and aortal pulse wave velocity (PWV) were assessed with SphygmoCor (AtCor Medical Pty Ltd., Sydney, Australia) using methods described in detail in our previous study [22]. The following parameters were analyzed: aortic (central) office systolic, diastolic, mean, and pulse pressure (AoSBP, AoDBP, AoMAP, AoPP (mm Hg)), augmentation pressure (AP = P2 − P1, where P2 is the amplitude of late, i.e., returning systolic peak pressure, and P1 is early systolic peak pressure (mm Hg)), augmentation index (AIx) expressed as AP divided by pulse pressure (AP/PP (%)), and P2/P1 ratio (%), as well as AIx (AP/PP) normalized to heart rate of 75 beats per minute (AIx75HR (%)), and aortic (carotid–femoral) pulse wave velocity. PWV was presented as (m/s) and (Z-score) based on normative pediatric data [24].

All children underwent transthoracic two-dimensional (2D), conventional Doppler, and tissue Doppler (TD) echocardiography (ECHO) with M-mode assessment of left ventricular parameters and simultaneous recording of ECG in the second limb lead with Philips iE33, transducer S5-1 (Philips, Amsterdam, The Netherlands). The following parameters were evaluated using a classical echocardiography and conventional Doppler technique. In the end-diastolic phase: the interventricular septum transverse diameter (IVSDd) (mm), left ventricular diastolic diameter (LVDd) (mm), left ventricular posterior wall diameter (LVPWd) (mm)), left atrial transverse diameter (LAD) (mm), relative wall thickness (RWT) calculated as 2 × LVPWd divided by LVDd, left ventricular mass calculated from the Deveraux equation, and left ventricular mass (LVMI) were indexed according to DeSimone [g/m^2.7^] [25], as well as shortening and ejection fraction (SF, EF) (%), peak wave velocity in early and late diastole caused by atrial contraction (the E and A waves) (cm/s), and E deceleration time (Edt) [s]. The TD was used to assess the mean value of peak medial and lateral mitral annular velocity during early filling (E’) (m/s), the mean value of peak medial and lateral mitral annular velocity during late filling (A’) (m/s), E/E’ ratio, isovolumetric relaxation time (IVRT) [s], isovolumetric contraction time (IVCT) [s], and a maximum speed of the systolic wave (C’) (m/s). Left ventricular hypertrophy (LVH) was defined as LVMI ≥ 95c. for age and sex [26], abnormal RWT was defined as >0.42. Mildly reduced and reduced ejection fraction was defined as EF between 41% and 49% and EF ≤ 40%, respectively, according to the 2021 European Society of Cardiology guidelines [27].

### 2.6. Statistical Analysis

Statistica 13.0 PL software (TIBCO Software Inc., Palo Alto, CA, USA) was used for calculations. The normality of the distribution of the analyzed variables was assessed using the Shapiro–Wilk test. Normally distributed data were presented as mean ± standard deviation (SD) and non-normally distributed variables as median and interquartile range (Q1–Q3). Differences between data were tested using the U Mann–Whitney test. The relationship between two variables was analyzed using Pearson’s linear correlation or Spearman’s correlation rank, depending on the distribution. Multivariate analysis was performed using forward stepwise regression analysis. Parameters that correlated with arterial and heart damage markers with *p* < 0.100 in univariate analysis were included in the final model. Parameters that correlated with each other with r > 0.650 were excluded from regression models to avoid collinearity. Logarithmic transformation of non-normally distributed data was performed prior to the analysis. As NT-proBNP and cardiovascular complications of CKD are significantly correlated to GFR, the latter variable was forced into the final model. A *p*-value < 0.05 was considered statistically significant.

## 3. Results

### 3.1. Clinical Characteristics

Clinical characteristics of children included in the study are summarized in Table 1. Most of the subjects were in CKD grades 2 and 3, and congenital anomalies of the kidney and urinary tract (CAKUT) were the leading primary kidney pathology. Among seven patients in grade G5, six were chronically dialyzed: five were treated with peritoneal dialysis (PD), one was treated with hemodialysis (HD), and one child with eGFR of 13.45 mL/min/1.73 m^2^ did not receive renal replacement therapy yet. Arterial hypertension was present in 26 patients, usually treated with one antihypertensive drug, most commonly calcium channel blockers or angiotensin-converting enzyme inhibitors.

### 3.2. NT-proBNP and Biochemical Parameters

**Figure 2:**
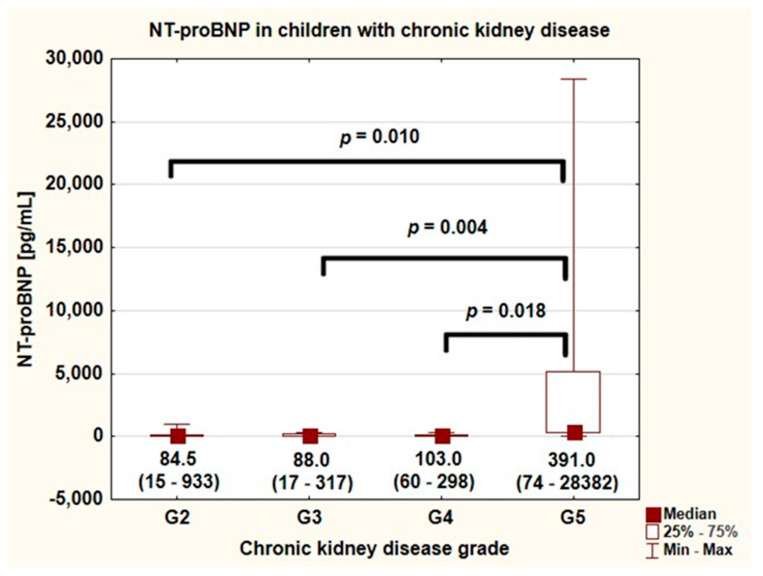
NT-proBNP in children with chronic kidney disease (median value and range) (G—chronic kidney disease grade).

The concentration of NT-pro BNP and remaining biochemical parameters are depicted in Table 2. The median value of NT-proBNP in patients with CKD was 95 (pg/mL) and varied from 15 up to 28,382 (pg/mL). NT-proBNP value above the norm (i.e., >125 pg/mL) was found in 16 (42.1%) children with CKD. NT-proBNP values did not differ significantly among children with CKD G2, G3, and G4. The highest values of NT-proBNP, significantly higher than children with CKD G2–G4, were found in children with CKD in stage G5 (Figure 2). In three of them, the NT-proBNP value exceeded 1000 (pg/mL). NT-proBNP at the concentration of 1579 (pg/mL) was detected in a 16.5-year-old boy with CKD and membranoproliferative glomerulonephritis, treated with hemodialysis. The boy had arterial hypertension treated with two drugs and left ventricular hypertrophy (LVH)—his LVMI was 40.2 g/m^2.7^. NT-proBNP at 5146 (pg/mL) was noted in a 14.5-year-old girl with CKD and steroid-resistant nephrotic syndrome treated with PD, with arterial hypertension treated with three drugs and LVH—her LVMI was 50.0 g/m^2.7^. The highest NT-proBNP concentration (28,382 (pg/mL)) was found in a 7.5-year-old female patient with unknown etiology of kidney disease, treated with PD, with arterial hypertension treated with two drugs, and LVH (LVMI—46.5 g/m^2.7^).

Relevant biochemical disturbances were found in the following numbers of CKD children—anemia in 13 (34%), hypercholesterolemia in 11 (29%), hypertriglyceridemia in 14 (37%), hypercalcemia in 1 (2.6%), hyperphosphatemia in 2 (5.2%), and elevated iPTH in 8 (21.1%) patients.

### 3.3. Blood Pressure and Markers of Arterial and Heart Damage

Blood pressure and markers of arterial and heart damage are shown in Table 3. At the time of the study, elevated office systolic blood pressure was found in 6 (15.8%) and elevated DBP in 4 (10.5%) children. Abnormal (i.e., ≥95c.) PWV was found in 1 (2.6%), and abnormal cIMT in 12 (31.6%) CKD children. LVH was found in 4 (10.5%) and abnormal RWT in none of the subjects. None of the children had mildly reduced or reduced EF.

### 3.4. Correlations of NT-proBNP and Markers of Arterial and Heart Damage

Significant correlations of NT-proBNP are depicted in Table 4. In CKD children, NT-proBNP correlated positively with markers of arterial damage: AP/PP, P2/P1, cIMT Z-score, and with the marker of diastolic dysfunction—E/E’. NT-proBNP concentration correlated negatively with the alfacalcidol dose and GFR. No significant relations were found among NT-proBNP and blood pressure, PWV ((m/s) and Z-score), AIx75HR, LVMI, RWT, SF, EF, and E/A.

PWV correlated significantly with peripheral and central DBP (r = 0.417, *p* = 0.034 and r = 0.406, *p* = 0.04, respectively), LVPWd (r = 0.506, *p* = 0.010), LVM (r = 0.482, *p* = 0.015), and A’ (r = 0.467, *p* = 0.038); PWV Z-score correlated with heart rate (r = 0.519, *p* = 0.013), A (r = 0.600, *p* = 0.011), and E/A (r = −0.578, *p* = 0.015); there was also trend towards a positive correlation between PWV Z-score and triglycerides (r = 0.404, *p* = 0.503); AP/PP correlated positively with PTH (r = 0.383, *p* = 0.048), cIMT Z-score (r = 0.533, *p* = 0.016), and A (r = 0.495, *p* = 0.031); P2/P1 correlated positively with calcium (r = 0.489, *p* = 0.010), alkaline phosphatase (r = 0.452, *p* = 0.020), PTH (r = 0.423, *p* = 0.028), cIMT Z-score (r = 0.510, *p* = 0.022), A (r = 0.616, *p* = 0.005), and negatively with E/A (r = −0.511, *p* = 0.026); cIMT correlated also positively with triglycerides (r = 0.461, *p* = 0.016) and negatively with calcium (r = −0.392, *p* = 0.043); cIMT Z-score correlated positively with triglycerides (r = 0.546, *p* = 0.011) and with RWT (r = 0.573, *p* = 0.008), negatively with E’ (r = −0.543, *p* = 0.030) and C’ (r = −0.733, *p* = 0.001).

LVMI correlated positively with triglycerides (r = 0.377, *p* = 0.030) and uric acid (r = 0.370, *p* = 0.031); RWT with C’ (r = −0.466, *p* = 0.011); E/A with calcium (r = −0.38, *p* = 0.043), alkaline phosphatase (r = −0.623, *p* < 0.001), and triglycerides (r = −0.450, *p* = 0.016); A’ with number of antihypertensive medications (r = 0.508, *p* = 0.013); E/E’ with calcium (r = 0.466, *p* = 0.011); and C’ correlated positively with hemoglobin (r = 0.413, *p* = 0.026), and negatively with AP/PP (r = −0.455, *p* = 0.044) and P2/P1 (r = −0.489, *p* = 0.029).

The correlations of the analyzed parameters with age are presented in Supplementary Materials Tables S1 and S2. In the studied children, there was no significant association between age and NT-proBNP (r = −0.166, *p* = 0.320). Age correlated negatively with serum calcium, inorganic phosphate, alkaline phosphatase, and pH (r = −0.336, *p* = 0.039; r = −0.397, *p* = 0.014; r = −0.590, *p* = 0.001, and r = −0.459, *p* = 0.001, respectively), and positively with both peripheral and central systolic and diastolic blood pressures expressed in (mm Hg) (r = 0.388–0.493, *p* = 0.046–0.009); no significant associations were found between age and blood pressure Z-scores. Age correlated also positively with PWV (m/s) (r = 0.490, *p* = 0.011), but not with PWV Z-score (r = 0.090, *p* = 0.677), and negatively with augmentation indices (r = −0.396–−0.521, *p* = 0.040–0.005). Moreover, numerous cardiac dimensions (IVSDd, LVDd, LVPWd, LAD) correlated positively with age (r = 0.459–0.727, *p* = 0.012–< 0.001); LV mass was positively related to age, too (r = 0.703, *p* < 0.001). In addition, age correlated negatively with A and positively with E/A and E’ (r = −0.393, *p* = 0.035; r = 0.464, *p* = 0.011 and r = 0.438, *p* = 0.017, respectively).

In the multivariate analysis, logNT-proBNP was the only significant predictor of the cIMT Z-score (beta = 0.402, 95CI (0.082–0.721), *p* = 0.014), and P2/P1 (beta = 0.130, 95CI (0.082–0.721), *p* = 0.014).

## 4. Discussion

Chronic kidney disease and cardiovascular disease are conditions that inter-influence. In CKD patients, a gradual decline in GFR leads to overhydration and accumulation of uremic toxins. Besides fluid overload, CKD patients are exposed to numerous other traditional (hyperlipidemia, volume-independent arterial hypertension) and non-traditional, i.e., “uremia-specific” risk factors, such as malnutrition, calcium–phosphorus disturbances, anemia, and hyperhomocysteinemia. Together, they contribute to cardiovascular damage and significant shortening of estimated lifespan [1,28]. Thus, it is crucial to establish the individual CVD risk to stratify patients to particular risk groups, diagnose the disease early, improve the treatment process, and initiate cardio- and renoprotective measures. NT-proBNP is one possible biomarker of increased cardiovascular risk.

In our cohort of 38 children, we observed an abnormally elevated value of NT-proBNP in almost half of the individuals. As there is no final consensus on normal pediatric NT-proBNP values, we used the manufacturer’s range. Nir and Lam proposed slightly higher normal values of the marker in the pediatric population, but they used a different kit [29,30]. NT-proBNP accumulates during CKD because of impaired renal clearance [31,32]. In our study group, NT-proBNP correlated negatively with GFR, and a gradual increase in NT-proBNP following CKD grades was found. A high concentration of NT-proBNP may contribute to cardiac strain in CKD, indicating vascular system overload. NT-proBNP provided essential prognostic and diagnostic information on fluid overload and cardiovascular damage in adults with CKD [9,33,34], despite its strong relation to kidney function. It was proven that elevated NT-proBNP concentration is correlated two-fold with mortality risk [33]. NT-proBNP level indicating increased CVD risk in CKD population seems to be substantially higher in comparison to healthy people [9].

There are limited data on the usefulness of NT-proBNP as a marker of cardiovascular damage in pediatric CKD patients. A positive correlation between NT-proBNP concentration and E/A, left atrial diameter, and left ventricle hypertrophy (LVH) was reported in small pediatric CKD cohorts [11,12]. We have observed a positive correlation between NT-proBNP and the degree of diastolic dysfunction measured by tissue Doppler echocardiography. Similarly, Kim et al. outlined the correlation between NT-proBNP and E/E’ in adults, suggesting that NT-proBNP might be an early marker of diastolic dysfunction in CKD patients [34]. No correlation among LV mass, LV ejection fraction, and NT-proBNP was found in our children. We hypothesize that this might be a derivative of a relatively good kidney function (66% of the studied subjects were in CKD grade G2 or G3) and a low prevalence of LV hypertrophy in the analyzed cohort. Of note, none of the patients had even mildly reduced left ventricular ejection fraction. In turn, mild heart damage in our cohort could be explained by a low grade of renal impairment and good control of arterial hypertension. This is a significant difference compared to studies in adult patients with CKD and might explain the failure to demonstrate a statistically significant relationship between left ventricular mass, systolic function, and NT-proBNP in the studied children.

Arterial damage is the earliest indicator of cardiovascular disease in CKD children. Unique, uremia-related biochemical milieu leads to Mönckeberg’s arteriosclerosis characterized by intramural calcium–phosphorus deposition, the osteoblast-like transformation of fibroblast, and a high risk of stroke or myocardial infarction [35]. We found numerous correlations among arterial damage and heart dimensions and function parameters, suggesting a strict interplay between arterial and cardiac dysfunctions in these patients.

We have found positive correlations among NT-proBNP, vascular stiffness indicators (AP/PP, P2/P1), and cIMT. NT-proBNP concentrations (expressed as decimal logarithms) were also significant predictors of cIMT and P2/P1 in multivariate analysis. While cIMT is a well-established marker of cardiovascular disease, P2/P1 and its derivative—the augmentation index shows a weaker correlation with hard-end points than the gold standard of arterial stiffness—aortic pulse wave velocity [36,37]. Little is known about pathophysiological relations between intimal and medial thickening and the heart. Sasaki found no significant associations between cIMT or the presence of atherosclerotic plaques and NT-proBNP level [38]. On the other hand, Asian authors found a positive correlation between cIMT and the concentration of this biomarker in adults with CKD [10,39,40].

NT-proBNP influences adipocyte function and was found to be negatively related to total and LDL-cholesterol concentrations [41,42]. These data suggest that NT-proBNP may have protective actions against arteriosclerosis and atherosclerotic plaque formation. It is possible that this compensatory mechanism is ineffective in CKD despite NT-proBNP accumulation. Our results suggest that NT-proBNP might serve in CKD pediatric patients as a valuable tool assessing the risk of arterial damage. Because of the cross-sectional study design, a causal relationship between cIMT thickening and NT-proBNP rise cannot be established. There is a need for prospective studies to establish its position as a biomarker of cIMT in this and other high-risk pediatric populations.

Numerous associations between NT-proBNP and calcium–phosphate metabolism parameters were observed in our study group, suggesting cardiovascular damage induced by these metabolic derangements. Similar relations were revealed in research conducted among pediatric CKD G3–G5 patients by Rinat [11]. In both adult [43] and pediatric [11,12] studies, NT-proBNP correlated positively with blood pressure. Despite evaluation of both peripheral and central blood pressure, no such relation was revealed in our cohort. We think that relatively mild kidney impairment and the common use of antihypertensive medications could mask this relationship. Furthermore, we evaluated blood pressure based on individual office measurements, which could be a source of potential bias.

Moreover, one should remember that, according to literature data, other factors may influence NT-proBNP levels, such as anemia, BMI (especially obesity), and gender [44,45,46]. None of these variables influenced NT-proBNP in our cohort, except for BMI. Nevertheless, this marker ought to be carefully interpreted in CKD patients considering factors that might affect it.

In our cohort of CKD patients, we revealed numerous significant correlations among biochemical parameters, heart dimensions, arterial stiffness parameters, and age. Of note, there was no significant association between age and NT-proBNP. A negative association between age and parameters of calcium–phosphorus metabolism reflects normal bone metabolism, varying with age, observed in both healthy [47] and CKD children [48]. As age and body size are crucial determinants of blood pressure and cardiac dimensions, proper indexation and comparison of the measured value with population-based norms is necessary in pediatric patients [21,26]. Noteworthy, age-normalized blood pressure and left ventricular mass index did not show any significant correlations with age.

Progressive stiffening of the arteries (measured as aortic PWV) is a well-known phenomenon, responsible, e.g., for isolated systolic hypertension in the elderly [49]. Age-dependent increase in PWV is seen already in the first two decades of life and was confirmed in large cohorts of healthy children [24,50]. On the other hand, the inverse relationship among age, body dimensions, and augmentation index was observed in the general population, similar to our cohort. In younger (and therefore shorter) patients, the pulse wave reflected from the peripheral arteries reaches back to the aorta more quickly due to its shorter pathway, resulting in an increase in the augmentation index in the youngest children, as revealed by Hidvegi et al. [51].

Some limitations of our study may be identified. We reported only a single-center study with a limited number of CKD patients, and broader research should be carried out, including a comparison of the NT-proBNP level in a control group. In addition, the vast majority of the subjects were in CKD G2 and G3 with minor biochemical and cardiovascular abnormalities, which might have influenced the number of NT-proBNP correlations. Finally, the study’s cross-sectional nature precludes drawing final casual relationships between NT-proBNP and the measured parameters.

## 5. Conclusions

Our cross-sectional analysis revealed numerous correlations between NT-proBNP and arterial and heart damage indices in children with chronic kidney disease. As NT-proBNP was a significant determinant of cIMT and P2/P1 in the multivariate analysis, we conclude that NT-proBNP may serve as a possible marker of thickening of the carotid artery wall in pediatric patients with kidney function impairment. NT-proBNP could be used in everyday clinical practice to assess cardiovascular risk in these subjects as evaluation of its serum concentration is easy accessible, relatively cheap, and repeatable. Conversely, due to our study’s limitations, the final role of NT-proBNP as a biomarker of arterial damage, left ventricular hypertrophy or diastolic cardiac dysfunction in children with CKD needs confirmation in prospective studies.

## Figures and Tables

**Table 1:** Clinical and biochemical data of the studied children.

Analyzed Parameter	Value (Mean ± SD or Median and Q1–Q3)
Age (years)	12.3 (8.6–16.3)
Gender (males/females)	21/17 (55%/45%)
CKD GRADE (n (%))	
G2	14 (37%)
G3	11 (29%)
G4	6 (16%)
G5	7 (18%)
Primary kidney disease (n (%))	
CAKUT	18 (47%)
Glomerulonephritis	7 (18%)
Hereditary nephropathy	3 (8%)
Toxic/ischemic kidney injury	3 (8%)
Cystic kidney disease	2 (5%)
Hemolytic uremic syndrome	1 (3%)
Other	1 (3%)
Unknown	3 (8%)
BMI Z-score	−0.1 ± 1.3
Overweight (BMI Z-score 1–2)	6 (16%)
Obesity (BMI Z-score >2)	2 (5%)
Underweight (BMI Z-score <2)	3 (8%)
Arterial hypertension	26 (68%)
Number of antihypertensive medications	1 (1–2)
Medications ^1^	
Angiotensin-converting enzyme inhibitor	16 (42%)
Angiotensin receptor antagonist	2 (5%)
Calcium channel antagonist	19 (50%)
Beta-adrenolytic	7 (18%)
Erythropoiesis-stimulating agents	11 (29%)
Vitamin D3	29 (76%)
Alfacalcidol	12 (32%)
Calcium carbonate	18 (47%)
Erythropoiesis-stimulating agents	11 (29%)

SD—standard deviation, Q1—the first quartile, Q3—the third quartile, CKD—chronic kidney disease, G—grade, CAKUT—congenital anomalies of kidney and urinary tract, BMI—body mass index. ^1^ number of patients.

**Table 2:** NT-pro BNP and biochemical characteristics of the study group (NT-proBNP—N-terminal pro-brain natriuretic peptide).

Analyzed Parameter	Value (Mean ± SD or Median and Q1–Q3)
NT-proBNP (pg/mL)	95.0 (52–298)
NT-proBNP G2 (pg/mL)	84.5 (32–140) ^1^
NT-proBNP G3 (pg/mL)	88.0 (44–174) ^2^
NT-proBNP G4 (pg/mL)	103.0 (72–171) ^3^
NT-proBNP G5 (pg/mL)	391.0 (317–5146) ^1,2,3^
Creatinine (mg/dL)	1.4 (0.9–2.4)
GFR (mL/min/1.73 m^2^)	43.7 ± 27.3
Urea (mg/dL)	45.0 (37.0–89.0)
Hemoglobin (g/dL)	12.4 ± 1.4
Albumin (g/dL)	4.4 (4.2–4.7)
cholesterol (mg/dL)	170.0 (157.0–208.0)
LDL-cholesterol (mg/dL)	96.0 (74.2–115.0)
HDL-cholesterol (mg/dL)	58.2 ± 16.9
Triglyceride (mg/dL)	101.0 (77.0–152.0)
Calcium (mg/dL)	10.0 ± 0.4
Inorganic phosphate (mg/dL)	4.7 ± 0.8
Intact parathormone (pg/mL)	53.5 (29.6–111.0)
Alkaline phosphatase (IU/L)	180.1 ± 77.6
25(OH)D (ng/mL)	21.2 (16.3–29.6)
Uric acid (mg/dL)	6.3 ± 1.3
pH	7.41 ± 0.04
HCO3^−^ (mmol/L)	24.6 (22.8–25.6)
BE (mmol/L)	−0.35 ± 3.16

SD—standard deviation, Q1—the first quartile, Q3—the third quartile, NT-proBNP—N-terminal pro-brain natriuretic peptide, GFR—glomerular filtration rate according to Schwartz formula, LDL—low-density lipoprotein, HDL—high-density lipoprotein, 25(OH)D—25-hydroxy-vitamin D, pH—power of hydrogen, HCO_3_^−^—bicarbonate, BE—base excess. ^1^ *p* = 0.010, ^2^ *p* = 0.004, ^3^ *p* = 0.018.

**Table 3:** Blood pressure, arterial, and heart parameters in the study group.

Parameter	Children with Primary Hypertension
Blood pressure and heart rate	
Peripheral office SBP (mmHg)	116.4 ± 12.9
Peripheral office SBP Z-score	0.99 ± 1.28
Peripheral office DBP (mmHg)	71.7 ± 12.7
Peripheral office DBP Z-score	0.82 ± 1.1
Peripheral office MAP (mmHg)	87.5 ± 12.3
Peripheral office PP (mmHg)	44.7 ± 7.7
Aortic office SBP (mmHg)	101.5 ± 13.9
Aortic office DBP (mmHg)	73.4 ± 12.8
Aortic office MAP (mmHg)	87.5 ± 12.3
Aortic office PP (mmHg)	27.4 ± 5.3
Heart rate [bpm]	82 ± 14.2
Arterial structure and function	
cIMT (mm)	0.47 ± 0.06
cIMT Z-score	1.77 ± 1.21
AP (mmHg)	1.5 (−1.3–5.3)
AP/PP (AIx) (%)	6.5 ± 16.2
P2/P1 (AIx) (%)	108.3 ± 25.4
AIx75HR (%)	12.4 ± 18.9
SEVR (%)	151.3 (139.3–173)
PWV (m/s)	4.56 ± 0.86
PWV Z-score	−0.37 ± 1.27
Heart structure and function	
IVSDd (mm)	6.0 (5–7)
LVDd (mm)	44.3 ± 7.0 (39–50)
LVPWd (mm)	6.0 (4.6–6.5)
LAD (mm)	28.2 ± 4.3
RWT	0.24 (0.22–0.28)
LVM (g)	79.8 (53.4–114.5)
LVMI (g/m^2.7^)	28.7 (26.4–33.3)
SF (%)	40.1 ± 5.8
EF (%)	70.5 ± 6.69
E (cm/s)	89.38 ± 13.43
A (cm/s)	59.97 ± 11.4
E/A	1.55 ± 0.37
Edt (ms)	165 (148–192)
E’ (cm/s)	13.11 ± 2.67
A’ (cm/s)	6.20 (5.5–6.5)
E/E’	6.94 (5.83–7.49)
IVRT (ms)	68.4 ± 22.2
IVCT (ms)	77.2 ± 19.58
C’ (m/s)	6.0 ± 1.2

SD—standard deviation, Q1—the first quartile, Q3—the third quartile, SBP—systolic blood pressure, DBP—diastolic blood pressure, MAP—mean arterial pressure, PP—pulse pressure, cIMT—common carotid artery intima media thickness, AP—augmentation pressure, P—peak pressure, Aix—augmentation index, AIx75HR—augmentation index normalized to heart rate 75 beats per minute, SEVR—subendocardial viability ratio, PWV—aortic pulse wave velocity, d—end-diastolic phase, IVS—interventricular septum transverse diameter, LVD—left ventricular diastolic diameter, LVPW—left ventricular posterior wall diameter, LAD—left atrial transverse diameter, RWT—relative wall thickness, LVM—left ventricular mass, LVMI—left ventricular mass index, SF—shortening fraction, EF—ejection fraction, E—peak wave velocity in early diastole, A—peak wave velocity in late diastole caused by atrial contraction, Edt—E deceleration time, E’—mean value of peak medial and lateral mitral annular velocity during early filling, A’—mean value of peak medial and lateral mitral annular velocity during late filling, IVRT—isovolumetric relaxation time, IVCT—isovolumetric contraction time, C’—maximum speed of systolic wave.

**Table 4:** Significant correlations of NT-proBNP with analyzed clinical, biochemical, and cardiovascular parameters in children with CKD (Spearman’s rank correlations).

Analyzed Parameter	R	*p*
Alfacalcidol dose (µg/24 h)	−0.365	0.043
Creatinine (mg/dL)	0.367	0.023
GFR (mL/min/1.73 m^2^)	−0.423	0.008
Urea (mg/dL)	0.407	0.008
Inorganic phosphate (mg/dL)	0.443	0.005
Intact parathormone (pg/mL)	0.435	0.006
Triglyceride (mg/dL)	0.492	0.002
AP/PP (AIx) (%)	0.451	0.018
P2/P1 (AIx) (%)	0.460	0.016
cIMT Z-score	0.504	0.020
E/E’	0.400	0.032

GFR—glomerular filtration rate, AP—augmentation pressure, PP—pulse pressure, Aix—augmentation index, P—peak pressure, cIMT—carotid intima-media thickness, E—peak wave velocity in early diastole, E’—mean value of peak medial and lateral mitral annular velocity during early filling by tissue Doppler.

## Data Availability

The data presented in this study are available upon request from the corresponding author.

## Supplementary Materials

The following are available online at https://www.mdpi.com/article/10.3390/jcm10194344/s1, Table S1: Correlations of age with NT-proBNP, clinical, and biochemical parameters in the study group. Table S2: Correlations of age with blood pressure, arterial, and heart parameters in the study group.
