# Supplementary material for: NT-proBNP as a Potential Marker of Cardiovascular Damage in Children with Chronic Kidney Disease
Source: J Clin Med. 2021 Sep 24;10(19):4344. doi: 10.3390/jcm10194344 (PMC8509500; doi:10.3390/jcm10194344)
Supplement: Supplementary file 1 [file jcm-10-04344-s001.zip › jcm-1358633-Supplementary Table S1.pdf]

Journal of
Clinical Medicine

MDPI

Supplementary Table S1. Correlations of age with NT-proBNP, clinical, and biochemical parameters in the study group.

Analyzed parameter	R	P
Age (years) vs. NT-proBNP(pg/mL)	−0.166	0.320
Age (years) vs. BMI Z-score	−0.248	0.133
Age (years) vs. Number of antihypertensive medications	0.123	0.463
Age (years) vs. Creatinine (mg/dL)	0.249	0.133
Age (years) vs. GFR (mL/min/1.73 m^2)	−0.047	0.781
Age (years) vs. Urea (mg/dL)	−0.044	0.792
Age (years) vs. Hemoglobin (g/dL)	0.045	0.790
Age (years) vs. Albumin (g/dL)	−0.007	0.969
Age (years) vs. Cholesterol (mg/dL)	−0.145	0.391
Age (years) vs. LDL-cholesterol (mg/dL)	−0.285	0.096
Age (years) vs. HDL-cholesterol (mg/dL)	0.119	0.478
Age (years) vs. Triglycerides (mg/dL)	0.044	0.798
Age (years) vs. Calcium (mg/dL)	−0.336	0.039
Age (years) vs. Inorganic phosphate (mg/dL)	−0.397	0.014
Age (years) vs. Intact parathormone (pg/mL)	0.016	0.928
Age (years) vs. Alkaline Phosphatase (IU/L)	−0.590	0.001
Age (years) vs. 25(OH)D (ng/mL)	−0.032	0.850
Age (years) vs. Uric acid (mg/dL)	0.199	0.230
Age (years) vs. pH	−0.459	0.005
Age (years) vs. HCO3- (mmol/L)	0.094	0.580
Age (years) vs. BE (mmol/L)	0.063	0.710

NT-proBNP—N-terminal pro-brain natriuretic peptide, GFR—glomerular filtration rate according to Schwartz formula, LDL—low-density lipoprotein, HDL—high-density lipoprotein, 25(OH)D—25-hydroxy-vitamin D, pH—power of hydrogen, HCO3-—bicarbonate, BE—base excess.
