# Supplementary material for: NT-proBNP as a Potential Marker of Cardiovascular Damage in Children with Chronic Kidney Disease
Source: J Clin Med. 2021 Sep 24;10(19):4344. doi: 10.3390/jcm10194344 (PMC8509500; doi:10.3390/jcm10194344)
Supplement: Supplementary file 1 [file jcm-10-04344-s001.zip › jcm-1358633-Supplementary Table S2.pdf]

Journal of
Clinical Medicine

Supplementary Table S2. Correlations of age with blood pressure, arterial, and heart parameters in the study group.

Analyzed parameter	R	P
**Blood pressure and heart rate**		
Age (years) vs. peripheral office SBP (mmHg)	0.388	0.046
Age (years) vs. peripheral office SBP Z-score	−0.096	0.634
Age (years) vs. peripheral office DBP (mmHg)	0.493	0.009
Age (years) vs. peripheral office DBP Z-score	0.286	0.148
Age (years) vs. peripheral office MAP (mmHg)	0.360	0.065
Age (years) vs. peripheral office PP (mmHg)	−0.070	0.728
Age (years) vs. aortic office SBP (mmHg)	0.310	0.116
Age (years) vs. aortic office DBP (mmHg)	0.490	0.009
Age (years) vs. aortic office MAP (mmHg)	0.360	0.065
Age (years) vs. aortic office PP (mmHg)	−0.368	0.059
Age (years) vs. heart rate (bpm)	−0.281	0.156
**Arterial structure and function**		
Age (years) vs. cIMT (mm)	0.311	0.115
Age (years) vs. cIMT Z-score	0.005	0.982
Age (years) vs. AP (mmHg)	−0.398	0.040
Age (years) vs. AP/PP (AIx) (%)	−0.474	0.013
Age (years) vs. P2/P1 (AIx) (%)	−0.521	0.005
Age (years) vs. AIx75HR (%)	−0.396	0.041
Age (years) vs. SEVR (%)	0.324	0.099
Age (years) vs. PWV (m/s)	0.490	0.011
Age (years) vs. PWV Z-score	0.090	0.677
**Heart structure and function**		
Age (years) vs. IVSDd (mm)	0.510	0.002
Age (years) vs. LVDd (mm)	0.727	<0.001
Age (years) vs. LVPWd (mm)	0.660	<0.001
Age (years) vs. LAD (mm)	0.459	0.012
Age (years) vs. RWT	0.286	0.101
Age (years) vs. LVM [g]	0.703	<0.001
Age (years) vs. LVMI ($g/m^{2.7}$)	−0.074	0.676
Age (years) vs. SF (%)	−0.088	0.651
Age (years) vs. EF (%)	−0.117	0.547
Age (years) vs. E (cm/s)	0.160	0.406
Age (years) vs. A (cm/s)	−0.393	0.035
Age (years) vs. E/A	0.464	0.011
Age (years) vs. Edt (ms)	0.007	0.971
Age (years) vs. E' (cm/s)	0.438	0.017
Age (years) vs. A' (cm/s)	−0.005	0.979
Age (years) vs. E/E'	−0.196	0.308
Age (years) vs. IVRT (ms)	−0.280	0.142
Age (years) vs. IVCT (ms)	−0.109	0.582
Age (years) vs. C′ (m/s)	−0.145	0.453

SBP—systolic blood pressure, DBP—diastolic blood pressure, MAP—mean arterial pressure, PP—pulse pressure, cIMT—common carotid artery intima media thickness, AP—augmentation pressure, P—peak pressure, AIx—augmentation index, AIx75HR—augmentation index normalized to heart rate 75 beats per minute, SEVR—subendocardial viability ratio, PWV—aortic pulse wave velocity, d—end-diastolic phase, IVS—interventricular septum transverse diameter, LVD—left

ventricular diastolic diameter, LVPW—left ventricular posterior wall diameter, LAD—left atrial transverse diameter, RWT—relative wall thickness, LVM—left ventricular mass, LVMI—left ventricular mass index, SF—shortening fraction, EF—ejection fraction, E—peak wave velocity in early diastole, A—peak wave velocity in late diastole caused by atrial contraction, Edt—E deceleration time, E′—mean value of peak medial and lateral mitral annular velocity during early filling, A′—mean value of peak medial and lateral mitral annular velocity during late filling, IVRT—isovolumetric relaxation time, IVCT—isovolumetric contraction time, C′—maximum speed of systolic wave.
